# Fever

**Published:** 1893-04

**Authors:** L. B. Anderson

**Affiliations:** Norfolk, Va.


### FEVER.

BY L. B. ANDERSON, M. D., NORFOLK, VA.

Animal heat arises from the union of oxygen in the blood with carbon, hydrogen, sulphur and iron, the molecular changes which occur in nutrition, whereby gases are changed to fluids, and fluids to solids, the mechanical action of blood corpuscles on each other, and on the tubes through which they circulate. The normal chemical chanes which are constanjly occuring in the mineral and organic constituents of the tissues and fluids under the influence of well-balanced vital forces, and the mechanical action of the muscular fibres of the heart and arteries in prope-ling the blood through their channels. In health, no matter how active and potential the producing agencies are, they are always counterpoised by the heat eliminating processes, viz : the breaking down of tissues and their conversion into fluids and fluids into gases—the elimination of carbon and other combustible matters by the lungs and liver, the secretion of mucous and other fluids from the mucous membrane, of sweat from the skin and urine from the kidneys, bearing away mineral and organic compounds, salts and acids, conversely, therefore, the suspension of one or more of the heat eliminating processes must result in an exaltation of temperature proportioned to the extent of such suspension.

The above mentioned physiological processes are purely vital, or, when chemical, under the control of vital laws. So soon as vital laws are suspended chemical laws come into exercise, old unions and combinations are dissolved and new bodies are formed. Complex as are the elements of which the organs and tissues of the animal economy are compared, so long as they are controlled by vital laws, chemical forces *per se*, are kept in abeyance and harmony reigns supreme. Let one organ or function be disturbed or suspended and the vital balances are thrown from their equipoise, and discordant vibrations indicate the loss. The nervous system no longer controlling the functions of circulation nutrition and secretion are suspended, and the products of retrogressive metabolism are retained in the system, to pass rapidly into new combinations, which are often acrid, irritating and exceedingly poisonous. When this condition obtains many of the heat producing agents being in more vigorous exercise than normal, while the heat eliminating functions are suspended, a rise of temperature will follow, but when in addition to these forces we have the rapid chemical changes which result from the commingling of such complex organic materials as are found in the retained secretions, a thermogenic laboratory is established, from which emanate gentle or tidal wave of caloric, according to the perversion of the vital laws, and the activity of the chemical forces, pervading the whole economy, constituting a condition we term FEVER.

Fever, therefore, is not an entity, but only an expression of some inward pathological condition, whether it be local inflammation or functional derangemement. And as itis impossible to conceive of the existence of disease in any organ whose organic and functional integrity is perfect, so it is impossible to conceive of a fever except as the result of some organic or functional aberration, when, therefore, a fever exists its source may always be traced to local inflammation or functional derangement, or both.

Fortunately for medicine on the one hand, all fevers arising from inflammation in certain organs and tissues, are readily traced to their source, and hence have not only assigned habitation, but a name. But unfortunately, on the other hand, all fevers arising from functional aberation have no designated source of origin or distinctive appellation by which their nature or pathology can be known. Hence, all fevers which are not expressions of local inflammation, such as pleurisy, peritonitis, gastritis, etc., or such as emanate from incubation of a specific virus, such as measles, variola, etc., are termed idiopathic, a name without scientific or pathological significance, and hence, calculated to obscure instead of elucidate the etiology, pathology and therapeutics of this constitutional disturbance. Such fevers as spring from inflammation, and its sympathetic disturbances, or such as arise from fatigue, shock, excitement, indigestible food, fermentation, etc., in the *prima via.*, or such as have their origin in the morbific impression of the virus of measles, small-pox, scarlet fever or other exanthematous troubles come not within the perview of this paper. But such fevers as are vulgarly known as "Bilious," "Malarious," "Bilio-gastric," "Bilio-gastro-enteric," "Miasmatic," "Marsh," "Autumnal" of every type, will briefly claim our attention. In order, however, to eliminate from the discussion all hypothetical, conjectional and imaginary matter, whether in etiology, pathology, or therapeutics, we will discard all consideration of so-called malaria, marsh, miasm, bacteria, micrococci, germs and bacilli, as pathological factors, or the basis of therapeutical indications, as too Utopian and visionary for a philosophical and practical essay.

INTERMITTENT, REMITTENT AND CONTINUED, COMPLEX FUNCTIONAL FEVERS—ETIOLOGY.

These fevers are essentially one and the same, in etiology, pathology and treatment. The intermittent type is the simplest and least complex, and is a key to the nature and treatment of the others. Often in the same family, we find cases with all these types, arising from the same causes and requiring only such variation in treatment as the complications, which modify the types, demand. They are developed in the strong as well as the weak, in the old as well as in the young, in the male and female alike. While they are more prevalent in certain badly drained, and hence wet countries, than in the higher, more rolling and dryer sections in certain seasons, they assail all classes and conditions, on the high, rolling, sandy hills of the piedmont, as well as those of the tide water. Heat and moisture are by no means essential to their production, for, as just stated, they exist far away from marshes and water courses, while in the Dismal Swamp of Virginia they are unknown, comparatively, in all seasons. Much cloudy and rainy weather in all localities, is a sure protection against the etiological factors of these fevers, while in long heated spells they prevail almost everywhere in or more of their types. Whille dampness has been esteemed a potent etiological factor in many diseases, its *modus operandi* has been an enigma. We will present, as briefly as possible, one phase of its morbific potency, which we have never seen or heard anywhere, which we believe will, when fully elaborated, throw a flood of light upon a hitherto obscure and inexplicable subject. Electricity, an all-pervading entity in nature, is composed of two principles, known as positive and negative. Two bodies charged with the same kind of electricity will repel each other—two charged with the two separately will attract each other. The earth is negatively electrified, the ether positively electrified. The water or moisture in contact with the earth becomes negatively electrified, and its molecules are repelled and they shoot up into the ether in the form of vapor. The matters, whether animate or inanimate, are thus deprived of their electricity, and the organs of the human system feel the loss. Thus when rapid evaporation is going on, for a day or two before a storm, persons subject to rheumatism, neuralgia, and even corns, are afficted with pains. In low, wet localities, where the sun's rays have free access, this evaporation and loss of electricity is constantly occurring with a loss of nervous energy, and a consequent enfeebled state of the organic functions. It will be readily seen what a potential factor this electrical mutation must prove in prostrating the vital powers, disordering the functions, impairing the secretions, unbalancing the circulation and generating disease.—(We can only indicate this much now, but it is a question pregnant with most important pathological problems.)

When one has been exposed for a time to the direct and reflected rays of a prostrating, sickening and often overpowering autumnal sun, alternated with cool evenings and nights, and rapidly raising vapors in the mornings, abstracting from the systhem the electrical energy so essential to its vigor, enfeebling the vaso-motor centres, rendering torpid the functions of the stomach, duodenum, liver, pancreas and bowels, the circulation as a consequence becomes languid, the secretions are suspended, catarrhal exudations line the mucous coatings of the alimentary canal, abstracting absorption and arresting secretion therein.

PATHOLOGY.

When this state of things obtains, and undue exertion, mental or physical shock, such as arises from overpowering grief, wounds, indigestion, parturition, diarrhœa, drinking of inpure water, sudden changes in the temperature, exposure to the sun, with the consequent rapid abstraction of electrical energy, you will so overpower the vaso-motor nerve centres so as to cause an accumulation of blood in the most enfeebled internal organs, it may be the brain, lungs, liver, stomach or bowels. If this abnormal determination of blood finds the vaso-motor nerves peculiarly languid, and hence the power of resistance in important vital organs very feeble, there will be such a profound accumulation and congestion as greatly to endanger the vital powers, constituting what is usually termed "congestive chill." If, on the other hand, when this centripetal determination occurs, should the vaso-motor nerve centres be possessed with a modicum of vital energy, this morbid accumulation will awaken demonstrative responses, and the temporarily enfeebled vessels will be excited to such active contractility as to hurl back the blood into its accustomed channels with such violent impetuousity as to excite an active fever, the duration of which may be measured by its violence, for, inordinately violent arterial excitement will, in obedience to a well-known physiological law, soon exhaust nervous irritability and muscular energy, and lapse into a corresponding period of repose. A period of languor, relaxation and repose follows, known as *intermission*. The vital functions having been thus rudely perverted, and as a consequence impaired secretions being retained, the equilibrium can be maintained for only a given time, because of the inability of the nervous centres to supply energy to the vessels under the depressing influences of the retained and vitiated secretions. Hence there will be an accumulation again in the least resisting internal channels, and another congestion and chill will occur. This rythmical condition may continue for a greater or less period, according to the greater or less ability of the vital organs to maintain their integrity.

While in some localities, under certain conditions, this condition usually termed chill and fever, may alternate with greater or less intervals for days and weeks, at other times a marked change in its type sooner or later occurs, often developed from the very first. In the prodrome of chill, often the integrity of the vital organs and tissues are so enfeebled and their secretions so perverted and morbific that their overpowering impression on the nervous centres prevents such a supply of energy to the congested vessels as will enable them to liberate themselves from their abnormal burthens Hence the reaction is more feeble, the paroxysm much longer, and the ordinary period of intermission is supplanted by only a greater or less decline of excitement, at a given time, to be speedily followed by another exacerbation, constituting a new type, denominated

REMITTENT FEVER.

When, however, the vital organs are greatly enfeebled, the secretions suspended, and the retained debris of retrogressive metabolism, pass under the control of chemical laws, acid matters are evolved, irritating the mucous lining of the stomach and bowls, suspending the functions of the liver and kidneys, and so overpowering the vital energies as to prevent even a feeble effort to restore the last equilibrium, and hence maintaining such a degree of excitement as to produce a

CONTINUED FEVER.

Such a fever may present every conceivable type, depending on the character and extent of the local aberrations, or the functional depravity of one or more organs. It may be a simple fever without any marked local manifestations; there may be oppression and congestion in one or more internal organs, such as the brain, lungs, liver, stcmach or bowels, constituting the congestive type of continued fever, or there may be inflammation in some one or more of the organ and tissues constituting the inflammatory type. Again, each of these types may be modified by the local inflammation of functional complication, so as to produce great prostration, constituting adynanic continued fever, or the nervous centres may be so affected in conjunction with the local tronbles or great functional depravity as to constitute every type of

TYPHOID FEVER.

Taking a review of the field we find a sudden suspension of the secretions and a prostration of the nervous centres resulting in chill and fever, which, being repeated at regular intervals, constitute intermittent fever. Then, when the system has been left to grapple with impaired secretions we find the the intermittent changes to remittent. And when the local troubles become more marked and the secretions get more depraved, we find the remittent assumes the continued type. And whether from a previously impaired condition of the functions persisting for some time, or the degeneration of the retained secretions, all the vital energies are profoundly impaired, we have a type of fever which is known as typhoid. Just here it is of momentous importance to direct attention to a morbid development in the progress of even a simple, uncomplicated, functional fever, and much more in a more violent continued fever, which explains a long train of morbid developments, which often change the type of the fever, and without a knowledge of which, philosophical treatment would be utterly impossible.

When from meteorological changes in the atmosphere, as previously disclosed, the vital functions are perverted, the secretions are suspended, and often matters are retained in the system, chemical laws come into play, and various alkaloids, known as ptomaines, or leucomaines are generated, presenting in their physiological action most of the poisonous alkaloids, known to the materia medica. This condition is most demonstrably disclosed in the advanced stages of tyiphod or adynamic fevers, when the vitiated secretions are suffered to accumulate in the small intestines, in which putrefaction is rapidly developed, leucomainic gases are evolved, tympanitis ensues, wild delirium, from the abortion of poisonous alkaloids, sets in and the already contaminated fluids of too distended intestinal vessels are passed into the seething and putrefying secretions until the bowels no longer able to hold them expel them with a rolling, rumbling ominous explosion, filling all the compartments with their horribly sickening and offensive ordors. These results of the decomposition of organic, nitrogenous matters, viz., organic alkaloids, often ensue in the early stages of simple functional fever, when improperly treated, and by their impress change a simple intermittent, into a remittent, or remittent into a continued, and an ordinary uncomplicated into a low typhoid fever, or, indeed, a putro adynamic fever.

To the philosophical and scientific physician, no subject in all the range of medical thought, can be of more interest or of more practical importance than this. Here is a case of wild delirium, without inflammation or congestion of the intercranial tissues, which Stokes fifty years ago intimated sprang from some morbid state of the bowels, but which now finds its solution in the presence of ptomaines. Thəse commotions from decomposed and decomposing accumulations in the bowels may be solid, liquid or gaseous. When either of the latter two they are readily absorbed, pervading all the organs and tissues, contaminating and poisoning the whole economy.

SYMPTOMS.

It is often the case in certain constitutions of the atmosphere that the functions while not demonstrably impaired or perverted, are greatly enfeebled, and the slightest draught upon the vital energies, such as a saline purge, will result in an overthrow of the balance of the circulation and an accumulation of blood will occur in the internal organs, the nose, feet and hands will become cold, sensations of cold streams running down the spine will be felt, becoming deeper and more pervasive, until the whole system trembles and shudders with the pervasive rigidity. It may last for only a few minutes, or may continue for an hour or more, while the cutaneous surface presents the appearance known as *cutis anserina*, the involuntary horripilation become so demonstrative as to throw the muscular system into the wildest agitation, agonizing pains shoot through the head and back, and all the limbs are bathed in agony, the loins feel as though they were distended almost to the point of separation, and the agitation is so great as to shake the bed, and often the furniture in the room, constituting a condition known as ague. The subject begs for more covering, even when loaded with every variety and quantity of bed and other clothing. He implores the bystanders to hug and hold him and to lie upon him. After awhile the face begins to flush, the hands and feet to become warm, the feeble pulse to become full and bounding, the whole system becomes blazing hot, the heart bounds with tremendous throbs, the whole arterial sytem is wild with unbridled excitement, the head aches as if it would burst, the thirst is insatiable, all the covering is thrown off, and ice and cold drinks are piteously besought for. These are the more demonstrative symptoms of ague and fever.

TREATMENT.

So soon as the ague begins, thirty drops of laudanum given at once will prevent the wild agitation and racking pains which usually accompany an ague. But it is only safe to give it when it is clear that it is a case of uncomplicated ague, which can be determined only after one or more paroxysms. If the bowels have not been well opened with a chola gogue cathartic so soon as reaction occurs, give ten or twelve grains of calomel and eighteen or twenty-five grains of jalap, and small doses of ipecac and aconite, with nitrate of potash, may be given every hour. crushed ice, ice buttermilk, etc., given almost *ad libitum.* Generally, in four or five hours the cathartic will act, bringing away large stools of black tary bile, commingled with feculent and mucous accumulations. The functions of the skin and kidneys are restored and the fever ceases. Now is the time to give all possible energy to the enfeebled vaso-motor nerves by giving in rational doses a safe and reliable neurasthenic. We say in *rational* doses, because, if given in too small quantities, you only increase, instead of arresting, another paroxysm, and if given in large doses you produce great prostration, which willl endanger congestion and hopeless prostration. To a man then, five grains of quinine every two hours till four doses are given, will amply protect the system from another invasion, while one or two similar doses per day for three or four days will generally suffice to maintain a valid convalescence.

REMITTENT AND CONTINUED FEVERS.

There are usually no difference in the premonitory symptoms of these and intermittent fevers. Their approach is generally more insidious and less demonstrative. A feeling of malaria may exist for several days, especially about noon. The bowels are more or less abnormal, either too loose or constipated ; slight headache, pains occasionally in the head, back and limbs, tongue furred and stickey, occasionally pointed and red at the tip ; appetite variable, with either a loathing of food or a morbid craving for it ; at certain periods of the day an overpowering heaviness and sleepiness, which cannot be overcome, is experienced.

In this condition any unusual impression upon the system will disturb the equilibrium of the sanguinous and nervous systems, and a chill, not as demonstrative as in the type already described, but a sense of coldness, accompanied with aching in the head and limbs, thirst, nausea, prostration and fever. The febrile excitement, like the chill, is not so violent, but is equally as pervasive and persistent. It will generally rise to its maximum height in the afternoon or early night, and about two o'clock in the morning begins to decline, and about seven or eight o'clock has reached its lowest stage. Then some coldness about the fingers, toes and nose, presages another paroxysm of fever similar to the first. If in the meantime, vitiated secretions have been permitted to accumulate in the *period via*, poisonous leucomaines are evolved and new and more serious complications are established. The alvine discharges are either too light colored or too dark, too hard and dry or too watery, emitting exceedingly offensive odors ; the skin is dry, the urine scanty and high colored ; the tongue more pointed and red, and the heat about the chest, head and abdomen, on the rise of fever, becomes more exalted. The remissions are less distinct ; the secretions are more scanty and depraved, the mind becomes more bewildered, and the vital forces more prostrated. If the morbid chain thus being forged be not speedily broken and healthful functional activities restored, another important link in the morbid chain will be forged, and all the adynamic symptoms will become much more conspicuous. The mental faculties are much more disturbed, the heat becomes more caustic, the tongue redder, dryer and more pointed with the papillae at the point enlarged, while the edges are red and sleek. The heat about the head, chest and abdomen, is pungent, and though the heat is so great, and the mouth is so dry, there is no corresponding thirst. The condition of the stomach and bowels is very different in different subjects, but there is at this stage, almost always an absence of bile and the presence of mucous in the alvine discharged. The abdomen, as above stated, is generally full, sometimes doughy, tympanitic and tender. The urine is scanty and high-colored, and its passage accompanied with a burning, and a most peculiar garlicky odor.

(*To be Continued.*)

